# A wider scope on the treatment of atrial fibrillation

**DOI:** 10.1007/s12471-012-0266-x

**Published:** 2012-03-06

**Authors:** J. R. de Groot

**Affiliations:** Heart Center, Department of Cardiology, Academic Medical Center/University of Amsterdam, Meibergdreef 9, 1105 AZ Amsterdam, the Netherlands

Atrial fibrillation (AF) is the most common chronic arrhythmia, and its incidence and prevalence are expected to double within the forthcoming decades [1–3]. Currently, approximately 250,000 patients have AF in the Netherlands, corresponding to a prevalence of 5.5% in patients older than 55 years [4]. The vast majority of those patients can effectively be treated with pharmacological rate or rhythm control. However, there is a small subset of patients that remains severely symptomatic despite treatment with class 1 or 3 antiarrhythmic drugs. For those patients, an invasive approach can be indicated [5, 6].

Historically, this means either a classic Cox-Maze 3 operation or a catheter isolation of the pulmonary veins. The Maze operation, although associated with impressive success rates in some centres, has been abandoned as stand-alone procedure because of its surgical complexity and the requirement of cardiopulmonary bypass. Catheter ablation for atrial fibrillation is less invasive and is being performed by an increasing number of operators and centres. The procedure has a lower efficacy, particularly in patients with persistent AF or an enlarged left atrium. Moreover, patients are frequently not free of AF after a single procedure, and more than one procedure might therefore be required. In a recent meta analysis, the single procedure success of catheter ablation for AF was 57%, which rose to 71% after multiple procedures in selected patients [7]. The 5-year freedom of AF rates from Bordeaux, one of the most esteemed AF ablation centres in the world, were 29% after a single procedure (40% after one year), which increased to 63% after up to 7 procedures [8]. The volume of the number of catheter ablations for AF in comparison with the number of patients with AF is limited: approximately 2200 catheter ablations for AF were performed in the Netherlands in 2010 (exact data from two centres missing, S.A.I.P. Trines, personal communication), accounting for less than 1% of the number of patients with AF [4].

Bearing this in mind, a thoracoscopic surgical approach toward pulmonary vein isolation was developed in an effort to combine the efficacy reported with surgical ablation with a less invasive approach. There are several small studies showing the feasibility and safety of thoracoscopic pulmonary vein isolation, and a recent systematic review demonstrates that absence of AF recurrence (without the use of antiarrhythmic drugs) is 79% in paroxysmal AF and 69% in persistent AF after a single procedure in the studies published so far [9]. The number of patients and the number of studies are low, but there also seems to be a tendency toward better outcomes when the procedures are performed in a hybrid fashion, that is, by a surgeon and electrophysiologist together. Data from Maastricht and from our own hospital show that in a mixed population of patients with paroxysmal and persistent AF 83 and 86%, respectively, are free of AF without the use of antiarrhythmic drugs one year after the procedure [9–11]. This suggests that a hybrid procedure, where the ablation lines are controlled for conduction block during the procedure, is associated with less AF recurrence.

Thoracoscopic or minimal invasive surgery for AF has not been established in the Guidelines as a ‘reasonable alternative’ for either antiarrhythmic drugs or catheter ablation, which might be due to the limited availability of published evidence. The most recent European Society of Cardiology (ESC) Guidelines for AF award a 2B recommendation for stand-alone minimally invasive surgery for AF only for patients with a previously failed catheter ablation [5]. The 2007 Heart Rhythm Society (HRS)/European Heart Rhythm Association (EHRA)/European Cardiac Arrhythmia Society (ECAS) Consensus provides three recommendations: surgery for AF can be considered in patients with a failed catheter ablation, in those with a contraindication for catheter ablation and in patients with a preference for surgery [6].

Therefore, investigators from Nieuwegein and Barcelona conducted the FAST study, a two-centre randomised trial, which was published in the first 2012 issue of Circulation. [12] The study included 124 patients (81% male, mean age 56 years) with a previously failed catheter ablation (67%) or with enlarged atria (>40 mm in the presence of hypertension, or >45 mm without hypertension, 33%). Patients were randomised to a redo catheter ablation or to a totally thoracoscopic surgical ablation, which consisted of pulmonary vein isolation with a bipolar clamp device with or without the addition of left atrial lines, constructed anatomically with a bipolar cooled rail device at the discretion of the operator.

Exclusion criteria included left atrium size >65 mm, left ventricular ejection fraction <45%, valvular disease, myocardial infarction, cardiac catheter or surgical procedures in the previous 3 months.

Atrial fibrillation was paroxysmal in 65% and persistent or long-standing persistent in the remainder. Follow-up was performed at 3, 6 and 12 months following the procedure with a 7-day Holter after 6 and 12 months. After one year of follow-up, atrial fibrillation was absent (without the use of antiarrhythmic drugs) in 36.5% of patients who underwent catheter ablation versus 65.6% of patients undergoing thoracoscopic surgery. The percentages were, of course, higher when the use of antiarrhythmic drugs was not taken into account: 42.9% for the catheter ablation group and 78.7% for the surgical ablation group.

The number of adverse events was similar between the two groups during follow-up, but there were significantly more periprocedural complications in the surgery group versus the catheter group. The most common complications were directly related to the nature of the surgical procedure: a pneumothorax occurred in six patients, a haematothorax in one, there was one sternotomy for bleeding and one pneumonia. Two patients in the surgical group received a pacemaker versus none in the catheter group. On the other hand, in each group one patient suffered from a transient ischaemic attack and one from pericardial effusion/tamponade.

The FAST trial sheds new light on the invasive treatment of AF by performing the first head-to-head comparison between catheter ablation and surgical ablation in patients with a previously failed catheter ablation or enlarged left atrium with or without hypertension. The study makes it clear that in this population surgical ablation of AF is clearly more effective than catheter ablation. The increase in efficacy, however, goes at the cost of more periprocedural complications. Most complications reported, however, were transient in nature, and it can be debated whether a pneumothorax should be counted as a complication at all in a procedure that relies on bilateral collapse of the lungs. It can be expected that the complication rate will fall with increasing operator experience [12].

With regard to the disappointing efficacy of catheter ablation, it should be noted that this was not a standard AF ablation population. At best, it could be considered the worst half of AF ablation patients since all patients had failed to respond to catheter ablation at least once before. On the other hand, before the index procedure, two-thirds of the patients had normal sized left atria and atrial fibrillation was paroxysmal in two-thirds. These factors are associated with a favourable outcome both for catheter ablation and for surgical ablation.

Should all patients with AF now be referred to the surgeon? It is really too early to tell, but it seems justified to apply HRS consensus criteria and take patient preference into account when deciding on an invasive approach. The FAST-2 study (NCT01336075) that randomises patients with paroxysmal AF between catheter and surgical ablation as first-line therapy is ongoing, and will further delineate the position of thoracoscopic surgery for AF.

The conclusions of the FAST study have not entered the Guidelines yet, but the scope on invasive treatment of AF has now become wider, and thoracoscopic surgery might prove a valuable alternative for catheter ablation, particularly for those patients who failed catheter ablation before.
